# Insights into the molecular regulation of monolignol-derived product biosynthesis in the growing hemp hypocotyl

**DOI:** 10.1186/s12870-017-1213-1

**Published:** 2018-01-02

**Authors:** Marc Behr, Kjell Sergeant, Céline C. Leclercq, Sébastien Planchon, Cédric Guignard, Audrey Lenouvel, Jenny Renaut, Jean-Francois Hausman, Stanley Lutts, Gea Guerriero

**Affiliations:** 1 Environmental Research and Innovation Department (ERIN), Luxembourg Institute of Science and Technology (LIST), L-4362 Esch/Alzette, Luxembourg; 2 Groupe de Recherche en Physiologie Végétale (GRPV), Earth and Life Institute - Agronomy (ELI-A), Université catholique de Louvain (UcL), 1348 Louvain-la-Neuve, Belgium

**Keywords:** Gene expression, Hemp, Hypocotyl, Laccase, Lignan, Lignin, Monolignols, Peroxidase, Proteomics

## Abstract

**Background:**

Lignin and lignans are both derived from the monolignol pathway. Despite the similarity of their building blocks, they fulfil different functions *in planta*. Lignin strengthens the tissues of the plant, while lignans are involved in plant defence and growth regulation. Their biosyntheses are tuned both spatially and temporally to suit the development of the plant (water conduction, reaction to stresses). We propose to study the general molecular events related to monolignol-derived product biosynthesis, especially lignin. It was previously shown that the growing hemp hypocotyl (between 6 and 20 days after sowing) is a valid system to study secondary growth and the molecular events accompanying lignification. The present work confirms the validity of this system, by using it to study the regulation of lignin and lignan biosynthesis. Microscopic observations, lignin analysis, proteomics, together with in situ laccase and peroxidase activity assays were carried out to understand the dynamics of lignin synthesis during the development of the hemp hypocotyl.

**Results:**

Based on phylogenetic analysis and targeted gene expression, we suggest a role for the hemp dirigent and dirigent-like proteins in lignan biosynthesis. The transdisciplinary approach adopted resulted in the gene- and protein-level quantification of the main enzymes involved in the biosynthesis of monolignols and their oxidative coupling (laccases and class III peroxidases), in lignin deposition (dirigent-like proteins) and in the determination of the stereoconformation of lignans (dirigent proteins).

**Conclusions:**

Our work sheds light on how, in the growing hemp hypocotyl, the provision of the precursors needed to synthesize the aromatic biomolecules lignin and lignans is regulated at the transcriptional and proteomic level.

**Electronic supplementary material:**

The online version of this article (10.1186/s12870-017-1213-1) contains supplementary material, which is available to authorized users.

## Background

The monolignol-derived products lignin and lignans are important plant specialized (secondary) metabolites. They are involved in crucial events related to plant development, such as plant defence, growth regulation, sap conduction and erect growth habit. Lignin strengthens mechanically the xylem by impregnating the secondary cell wall of both tracheary elements and fibres. This phenomenon is particularly obvious in trees, but herbaceous plants also undergo lignification, particularly in the xylem of roots and the hypocotyl [1]. Lignans are formed by the stereospecific oxidative coupling of hydroxycinammyl alcohols mediated by a dirigent protein and an oxidase, typically a laccase [2, 3]. Their role in plant defence is known since more than a decade [3], but their plant growth regulatory activity is still under investigation [4]. Very few studies have addressed how this growth regulatory activity is mediated *in planta* [5, 6]. Lignin and lignans originate from the same building blocks, i.e. the monolignols, but have very different functions. Therefore, the allocation of the building blocks to either biosyntheses has to be precisely tuned, both temporally and spatially. As for the majority of genes involved in secondary cell wall deposition, the expression of lignin biosynthetic genes is regulated by master regulators belonging to the NAC and MYB transcription factor families [7]. By contrast, the expression of pinoresinol lariciresinol reductase (PLR), a key gene of lignan biosynthesis, was found to be higher in the young stem as compared to older stems in *Forsythia* x *intermedia* [8], suggesting that lignan biosynthesis may be somehow independent from secondary cell wall deposition. However, a role has been ascribed to lignans in secondary cell wall-forming tissues, where they may participate in the maintenance of the cell redox homeostasis during lignification [9, 10]. The genes coding for enzymes involved in lignan biosynthesis may thus be different according to the stage of development and tissue. This is illustrated by the expression patterns of the pinoresinol reductases *AtPRR1* and *AtPRR2*. The former is coexpressed with several secondary cell wall genes in the lignified internodes, while the latter is highly expressed in the growing hypocotyl [7]. Lignan and lignin biosyntheses may be intertwined, as a triple laccase mutant which displays strongly reduced lignin content shows a higher transcript level of *PRR2* [11].

Some dirigent proteins (DIR) are putatively involved in lignan biosynthesis, while others are related to lignin deposition. For example, AtDIR10/ESB1 plays an essential role in the formation of the Casparian strip in *Arabidopsis* by targeting lignin polymerization at specific extracellular sites [12] and AtDIR6 confers the (−) stereoconformation to pinoresinol [13]. The phylogenetic analysis of DIR helps to differentiate between those DIR involved in lignan biosynthesis (e.g. AtDIR6) and those to which other functions, such as lignin deposition, are assigned and referred to as DIR-like. According to Ralph [14], proteins of the DIR-a subgroup (AtDIR5, AtDIR6, AtDIR12 and AtDIR13) are wound- or insect-induced while proteins from the DIR-b subgroup are part of the constitutive defence of the plant. Since most of DIR have not yet been functionally characterised, phylogenetic analysis is a helpful tool that complements gene coexpression analysis for functional prediction [15].

Because lignin is racemic and since there is no mass spectral evidence about the occurrence of oligolignol stereoisomers, the proposed model of protein-driven control and template replication of lignin polymerisation [16], has failed to prove its robustness [17]. Therefore, the currently known functions of DIR are restricted to confer stereospecificity to lignans and to influence lignin deposition. Data from gene expression analysis during specific stages of development (stem elongation, secondary growth, xylem lignification) may contribute to get further details about their functions.

**Fig. 1:**
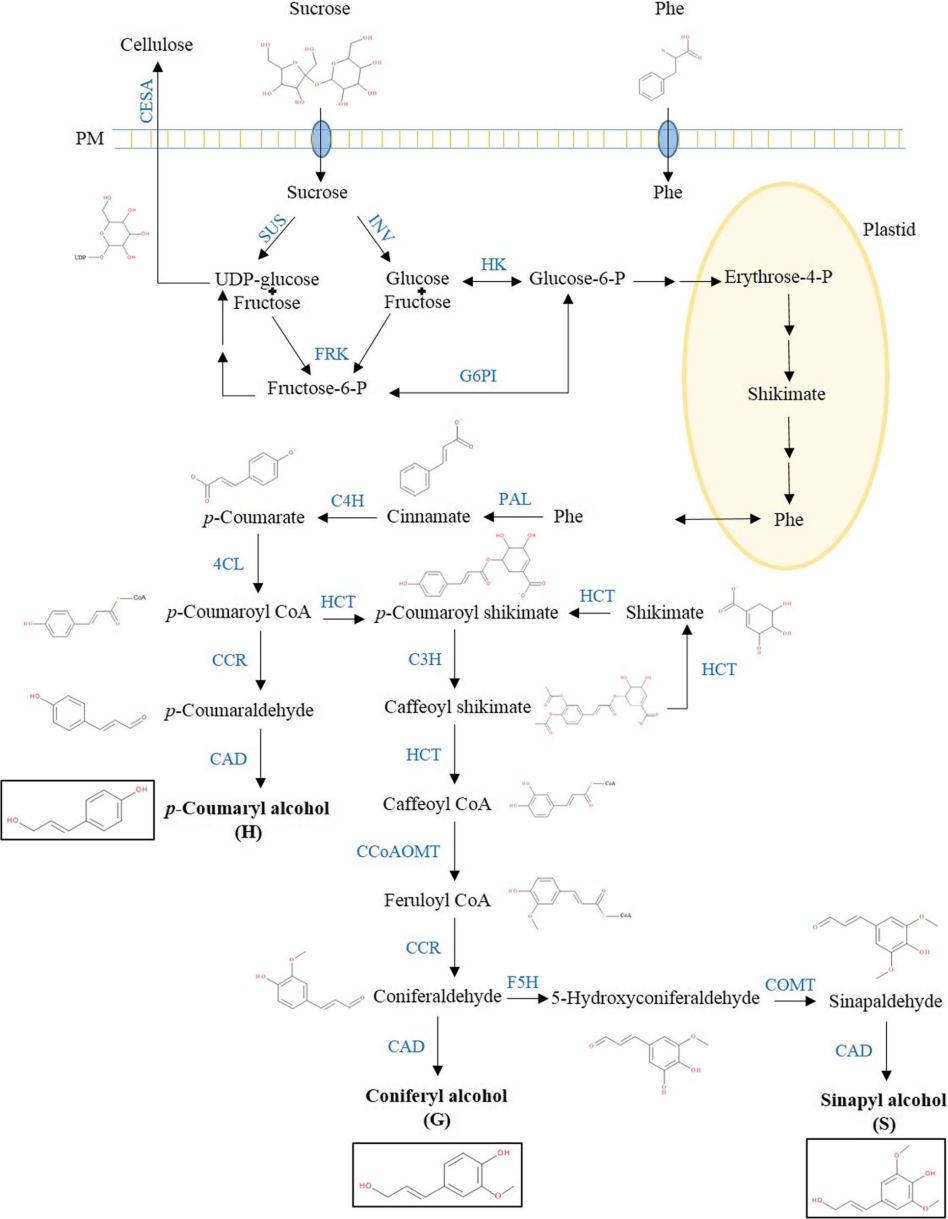
Monolignol and cellulose pathways. The molecules (in black) and enzymes (in blue) of the two pathways are indicated. Cellulose synthase (CESA) is membrane bound. Cinnamate-4-hydroxylase (C4H) and coumarate 3-hydroxylase (C3H) localise in the endoplasmic reticulum; hydroxycinnamoyl transferase (HCT) is partially associated with the endoplasmic reticulum [71]. All the other enzymes are active in the cytosol. 4CL 4-coumarate ligase, CAD cinnamyl alcohol dehydrogenase, CCoAOMT caffeoyl-CoA 3-O-methyltransferase, CCR cinnamoyl CoA reductase, COMT caffeate O-methyltransferase, F5H ferulate 5-hydroxylase, FRK fructokinase, G6PI glucose-6-phosphate isomerase, HK hexokinase, INV invertase, PAL phenylalanine ammonia lyase, Phe phenylalanine, SUS sucrose synthase

Following monolignol biosynthesis, lignin polymerisation is performed through end-wise radical coupling of phenols to the free-phenolic end of the growing polymer [17]. The formation of the radical is catalysed by either laccase or class III peroxidase and mainly takes place in the apoplast. In Angiosperms, the relative proportion of *p*-coumaryl (H), guaiacyl (G) and syringyl (S) units of the lignin polymer depends on the tissue, the stage of development and the subcellular compartment. Starting from phenylalanine, the biosynthesis of H, G and S units requires the activities of 5, 8 and 10 enzymes, respectively (Fig. 1).

In Angiosperms, the water-conducting cells of the xylem are enriched in G-lignin, while structural fibres (both from xylem and phloem) have a high S-lignin content [18]. Lignification begins with the deposition of the G units, notably in the secondary cell wall of xylem cells [19, 20], under the activity of laccases (LAC). H units are targeted to the middle lamella, while G units are initially deposited in the S1 sub-layer of both Gymnosperms and Angiosperms [16]. Less is known about the role of enzymes partaking in the oxidation and deposition of the lignin macromolecule. It has been suggested that laccases (LAC) and peroxidases (PRX) do not function redundantly in lignin polymerisation in the vascular tissues of *A. thaliana* [11]. Indeed, a knock-out mutant of *AtPRX52* has shown a decrease in the S units in the interfascicular fibres [21], suggesting that laccases do not compensate for the loss of peroxidase activity. By contrast, the lignin of *AtPRX2*, *AtPRX25* and *AtPRX71* knock-out mutants is richer in S units than wild type [22]*,* showing that a lower peroxidase activity is not synonymous to a decrease in S unit. The silencing of two genes coding for laccases (*AtLAC4*, *AtLAC17*) induces an increase in the fibre S/G ratio [19]. AtLAC17 is specifically involved in the deposition of G lignin in fibres, while the specific activity of AtLAC4 is less clear. More recently, the regulation of flax laccase expression by the microRNA *miR397* has been shown [23]. The question thus arising relates to the activity of these enzymes towards the oxidation of specific lignin subunits.

Between 6 and 20 days after sowing, the hemp hypocotyl was shown to be a suitable system to study the molecular events underlying secondary growth and secondary cell wall deposition [24]*.* The late stages of hypocotyl development are characterised by the up-regulation of transcription factors and genes involved in the synthesis of precursors needed for secondary cell wall deposition, the biosynthesis of monolignols and lignin polymerization. The same experimental set up is here used to study lignin biosynthesis: after chemical characterisation of lignin, the laccase and peroxidase activities are assessed. These results are put in perspective with gene expression and proteomics data. We also provide preliminary results dealing with putative orthologs involved in lignan biosynthesis, based on phylogenetic and targeted gene expression analyses.

## Results

### Time-course analysis of lignification in the hypocotyl

**Fig. 2:**
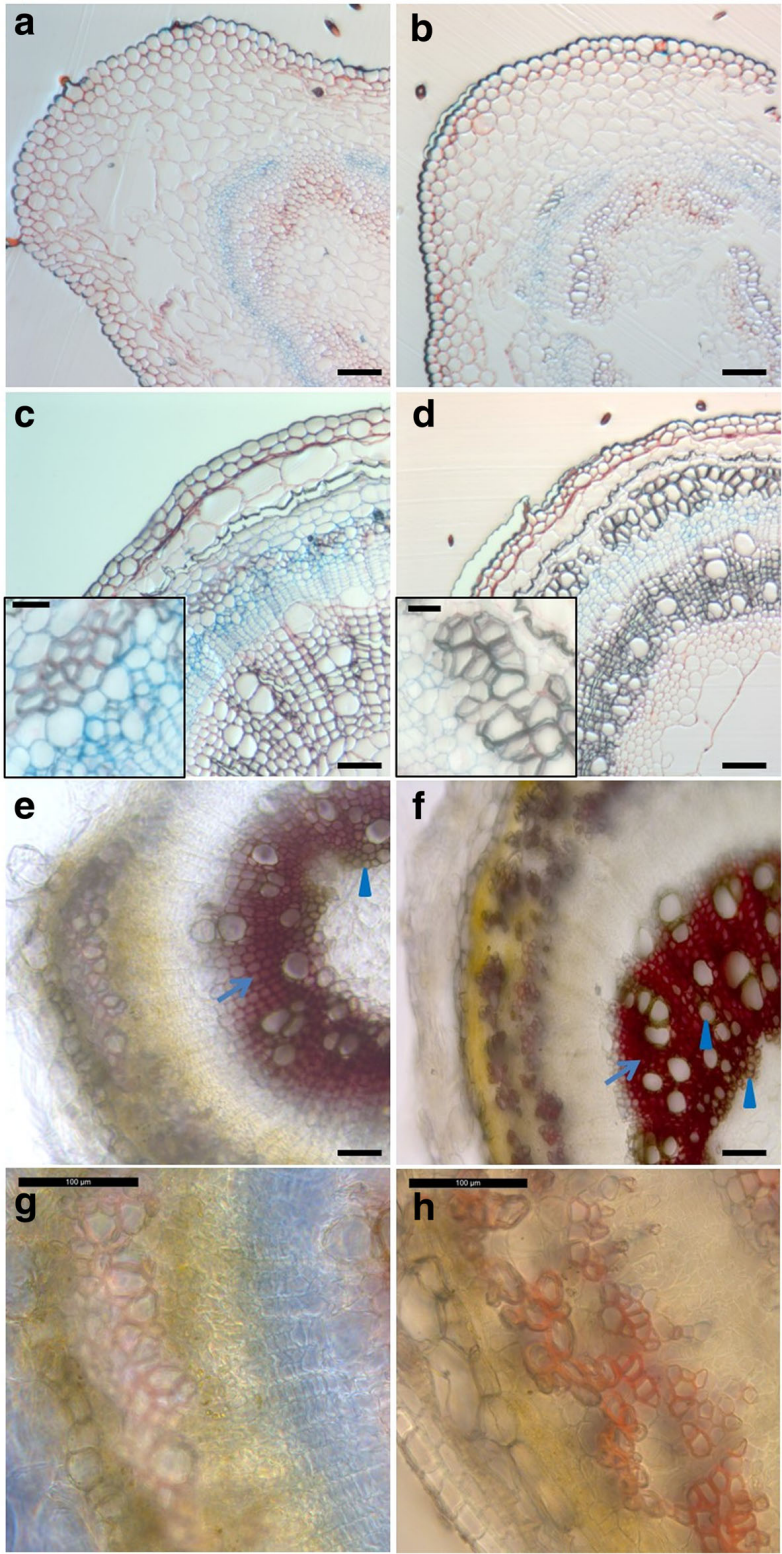
Lignification of the hemp hypocotyl between 6 and 20 days. **a** to **d**, fixed cross sections of H6 (**a**), H9 (**b**), H15 (**c**) and H20 (**d**) stained with FASGA. Lignification of the bast fibres is illustrated in the insets of **c** and **d**. **e** to **h**, fresh cross-sections of H15 (**e** and **g**) and H20 (**f** and **h**) stained with Mäule reagent. Higher magnifications of the bast fibres are shown in **g** and **h**. The primary xylem cells and tracheary elements of the secondary xylem are indicated with arrow heads, while fibres of the secondary xylem are indicated with an arrow in **e** and **f**. Scale bar = 100 μm in the main pictures and 25 μm in the insets

Hypocotyl lignification was followed between 6 (H6) and 20 (H20) days after sowing. The development was monitored by staining the cross sections of the four time points (H6, H9, H15 and H20) with a FASGA solution (a mix of Alcian blue and safranin). Alcian blue stains the cellulosic walls in blue, while safranin produces a red-orange coloration with lignin. In H6 and H9, the xylem cells are stained purple (Fig. 2a-b). In H15 and H20, the cambial cells display a strong blue coloration, together with a thin layer of the bast fibres (Fig. 2c-d, insets). A weak red edging is also visible in the cell wall of the bast fibres, indicating the presence of a small amount of lignin (Fig. 2c-d). The Mäule staining confirms the lignification of the bast fibres, with a red coloration in both primary (H15, Fig. 2e) and secondary bast fibres (H20, Fig. 2f). As expected, lignin is detected in all the xylem cells, both with FASGA and Mäule. The Mäule staining differentiates primary from secondary xylem cells based on the differential presence of syringyl (S) units in the lignin of these two tissues. In H15 and H20, primary xylem cells are stained brown, indicating that the lignin polymer lacks the S units; the fibres of the secondary xylem, as well as the bast fibres, are instead stained red indicating that they are rich in S lignin (Fig. 2e-f-g-h).

Lignin content was determined using the acetyl bromide method (Table 1). Significant differences were found across development, from 1.94% of the cell wall residue (CWR) in H6 to 4.70% in H20. The composition of the lignin was determined based on the quantification of products after nitrobenzene oxidation (NBO) of cell wall residue (Table 1). The three main lignin degradation products (*p*-hydroxybenzaldehyde, vanillin and syringaldehyde corresponding to H, G and S monolignol units, respectively) were recovered in all the samples. Since the proportion of H-units is an indicator of lignin condensation, the decrease in the H/H + V + S ratio (from 13% to 3%) may indicate that lignin is less condensed in older hypocotyls. The S/V ratio, by contrast, increased with the hypocotyl age (from 0.18 to 1.04).

**Table 1:** Lignin content and monomer composition of the hemp hypocotyl

	H6	H9	H15	H20
Lignin (% CWR)	1.94 (0.39) a	2.71 (0.48) a	4.54 (0.20) b	4.7 (0.19) b
H (μmol/g CWR)	9.72 (0.94) b	7.82 (1.40) ab	4.79 (0.73) a	5.24 (0.90) a
V (μmol/g CWR)	54.12 (8.19) a	73.07 (15.08) a	41.72 (7.14) a	84.93 (22.82) a
S (μmol/g CWR)	9.69 (0.95) a	17.41 (2.00) a	36.09 (7.98) ab	87.29 (23.52) b
S/V	0.18 (0.01) a	0.26 (0.03) a	0.85 (0.04) b	1.04 (0.03) c
H/(H + V + S)	0.13 (0.01) d	0.08 (0.00) c	0.06 (0.00) b	0.03 (0.00) a

Within a row, values with different letters are significantly different (Tukey *p*-value < 0.05). The values indicate the average of 3–5 biological replicates (see Methods section), with the standard error of the mean (SEM) in parenthesis. CWR cell wall residue, H *p*-hydroxybenzaldehyde, V vanillin, S syringaldehyde

### Gene expression analysis during hypocotyl lignification

**Fig. 3:**
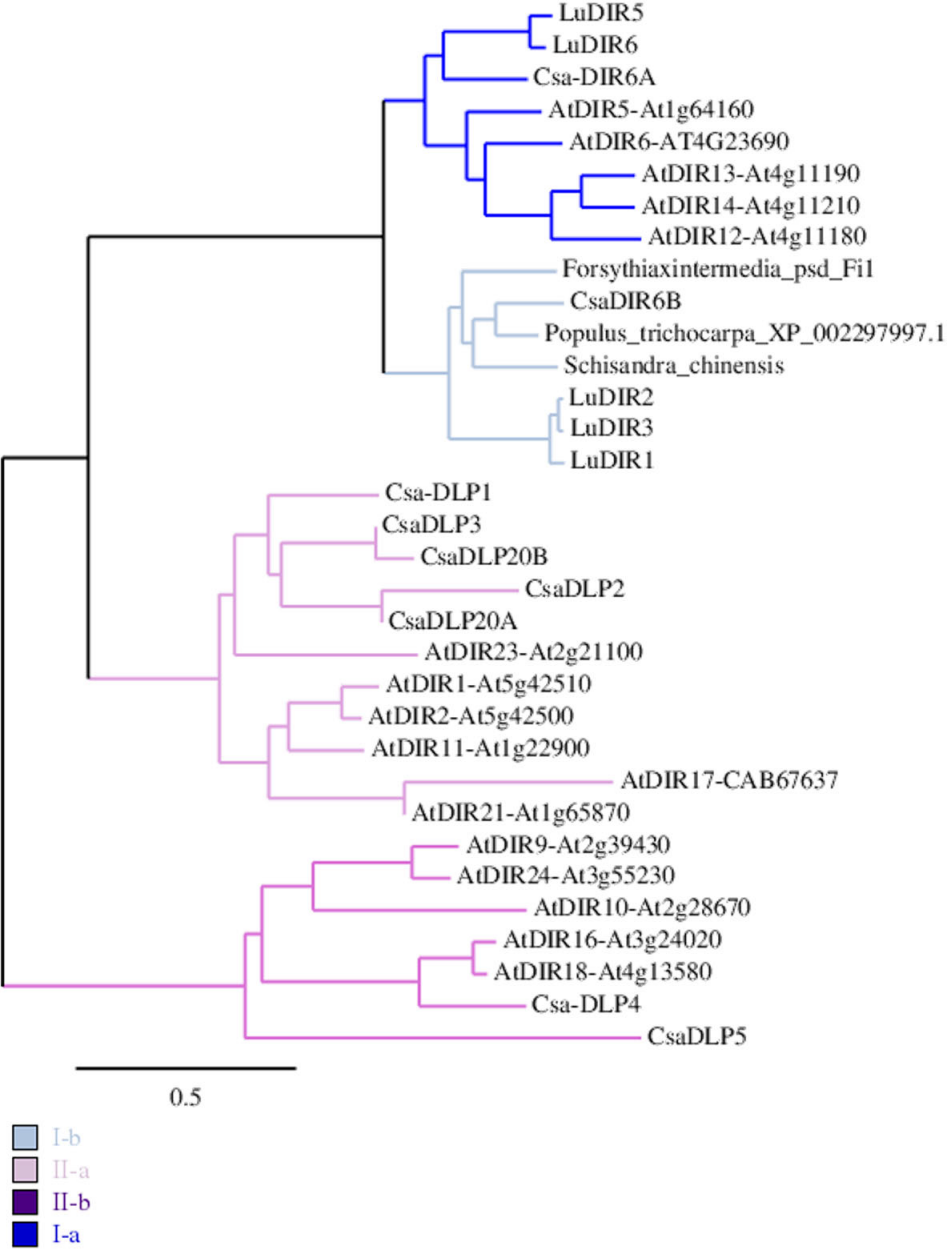
Phylogenetic analysis of DIR and DIR-like proteins (DLP). *Cannabis sativa* (Csa), *A. thaliana* (At), *Forsythia* x *intermedia*, *Populus trichocarpa, Schisandra chinensis* and *Linum usitatissimum* (Lu). Neighbour-joining tree calculated with 1000 bootstraps replicates with bioNJ algorithm (phylogeny.fr; [72]). Scale bar: expected numbers of amino acid substitutions per site. The sequences are in the Additional file 1

DIR have been divided into two subfamilies, namely DIR-a (DIR) and DIR-like (DLP, [14]), based on their sequences (Additional file 1) and possible biochemical functions. In order to predict a function for the dirigent proteins of *C. sativa*, a phylogenetic tree was built with DIR and DLP from *Arabidopsis*, *Linum usitatissimum, Forsythia* x *intermedia*, *Schisandra chinensis* and poplar [13, 14, 25, 26]. The tree is also available in the TreeBASE public repository. Two main clades appeared (Fig. 3). Clade I contains pinoresinol forming DIR from *Arabidopsis*, *L. usitatissimum*, *F. intermedia* and *S. chinensis* as well as two DIR from *C. sativa*, i.e. CsaDIR6A and CsaDIR6B. Furthermore, two sub-clades (I-a and I-b) respectively group (−)- and (+)- pinoresinol forming DP. Interestingly, CsaDIR6A belongs to subclade I-a while CsaDIR6B belongs to subclade I-b. Clade II contains the DIR-like proteins from *Arabidopsis*. The subclades II-a and II-b correspond to the DIR-d and DIR-e family described by Ralph and colleagues [14], respectively.

**Fig. 4:**
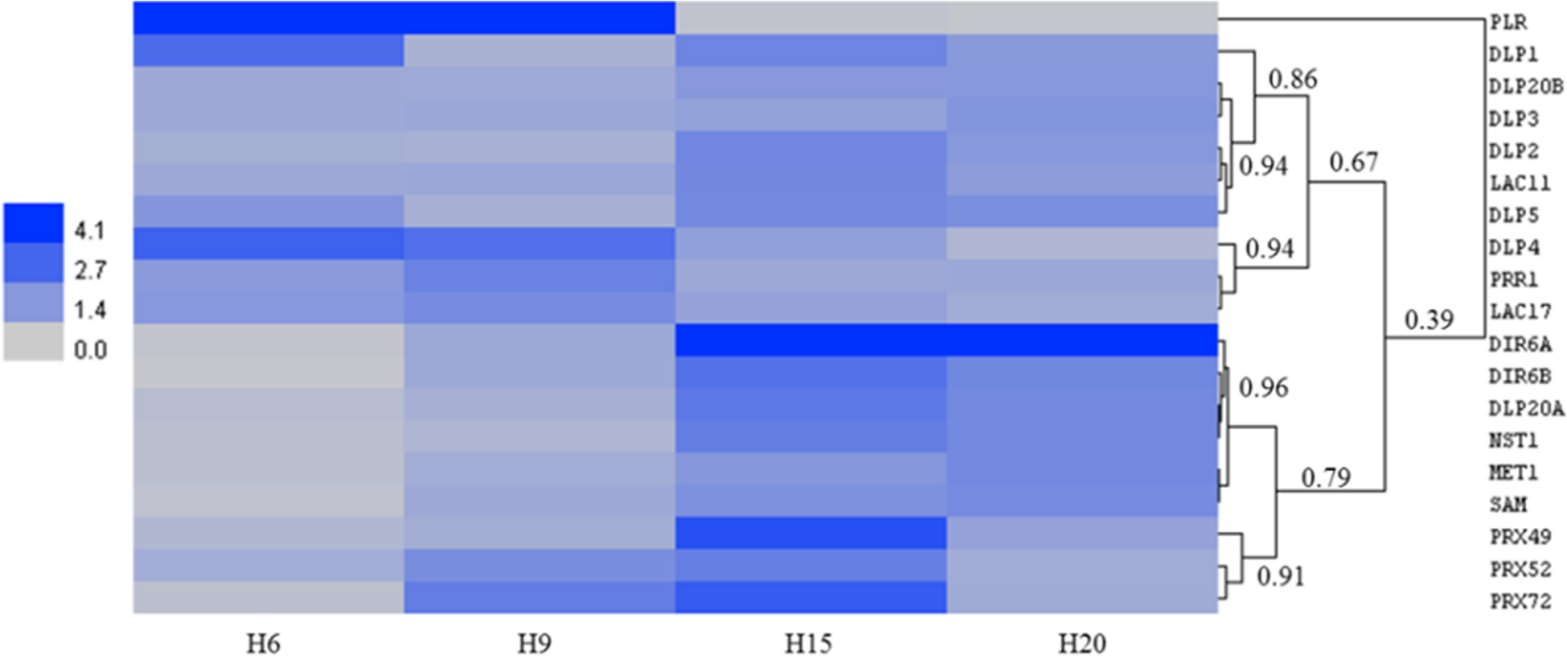
Heatmap hierarchical clustering showing the expression of genes assessed by RT-qPCR. Values represent Calibrated Normalized Relative Quantities (CNRQ) calculated with qbase^+^. DLP dirigent-like protein, LAC laccase, PRR1 pinoresinol reductase 1, DIR dirigent protein, NST1 NAC secondary cell wall thickening 1, MET1 methionine synthase 1, SAM *S*-adenosylmethionine synthase, PRX peroxidase, PLR pinoresinol-lariciresinol reductase. The colour bar indicates the expression values represented as an increasing intensity gradient. The numbers refer to the Pearson correlation coefficients between the lengths of two branches. The CNRQ data are given in Additional file 2

Two patterns of gene expression are observed based on the hierarchical clustering (Fig. 4 and Additional file 2). With the exception of *DLP20A*, all the DIR-like genes branched in the top cluster (*DLP1*, *DLP20B*, *DLP3*, *DLP2*, *DLP5* and *DLP4*). Their corresponding proteins belong to clade II of the phylogenetic analysis (Fig. 3). *DLP20A*, together with *DIR6A* and *DIR6B* (clade I of the phylogenetic tree), were part of the bottom cluster. Genes of the top cluster were either more expressed in H6 or H9 (*DLP1*, *DLP4*, *PRR1*, *LAC17*), or showed no major changes in their expression. PLR was most expressed in H6 and H9. The opposite trend was observed for the bottom cluster: genes involved in secondary cell wall biogenesis (*NST1*, *MET1*, *SAM*, *PRX49*, *PRX52* and *PRX72*), *DIR6A*, *DIR6B* (based on sequence clustered with proteins involved in pinoresinol biosynthesis) and *DLP20A* were more expressed in H15 or H20. Two trends of expression were thus observed for the DIR and DLP, which may point to different physiological roles.

### Proteomics analysis by gel-based and gel-free methods

Soluble proteomes from H6, H9, H15 and H20 have been analysed in 5 biological replicates by gel-based and gel-free methods.

**Fig. 5:**
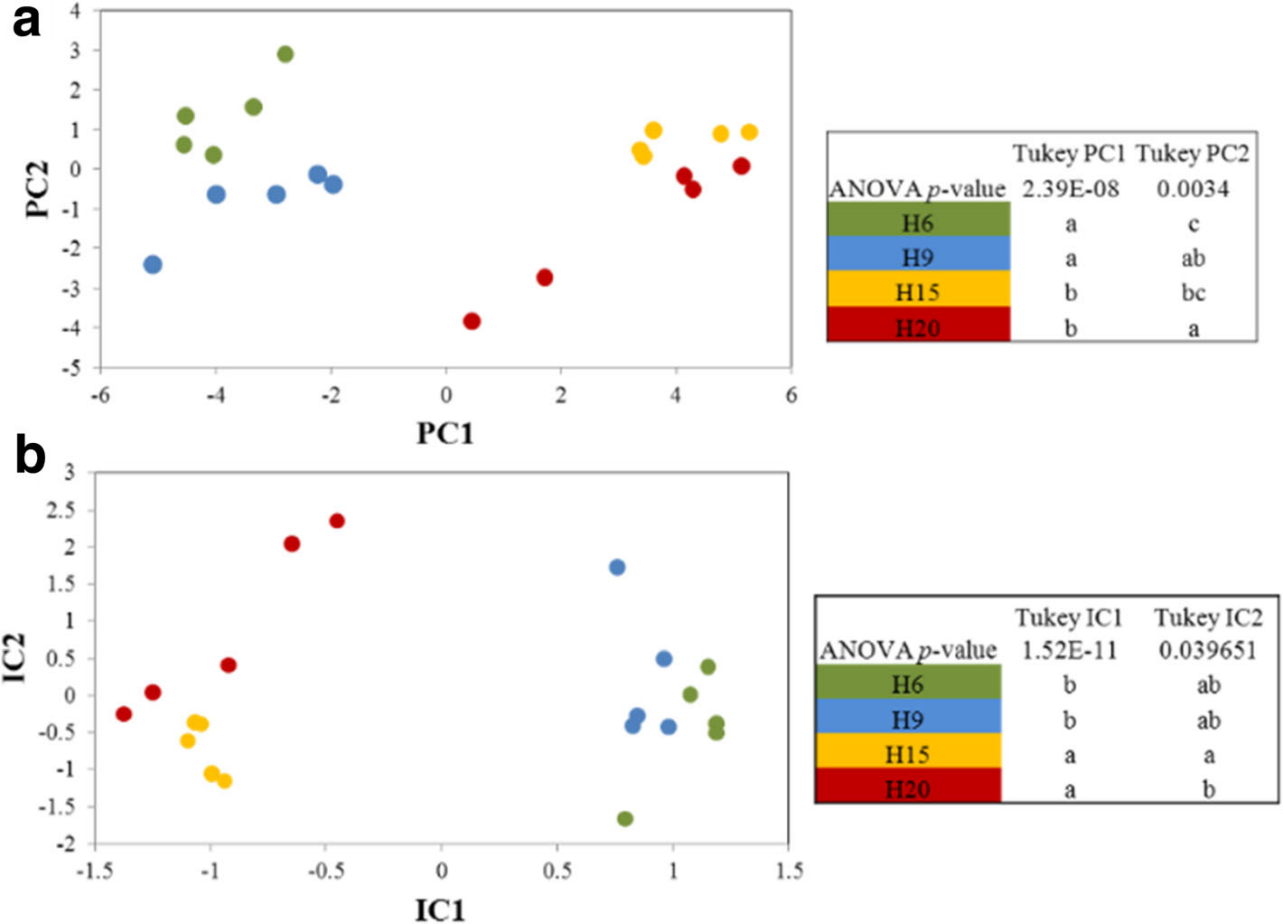
Clustering of the proteome profiles of hypocotyls at different ages**.** H6 green dots, H9 blue dots, H15 orange dots, H20 red dots. **a**: Principal component analysis based on the gel-based proteome study. **b**: Independent component analysis of LC-MS/MS based proteome profiles. In both panels, the significance of the coordinates in the two main axes was assessed using a Tukey post-hoc test, different letters within one column indicate that the proteome profiles are significantly different

**Fig. 6:**
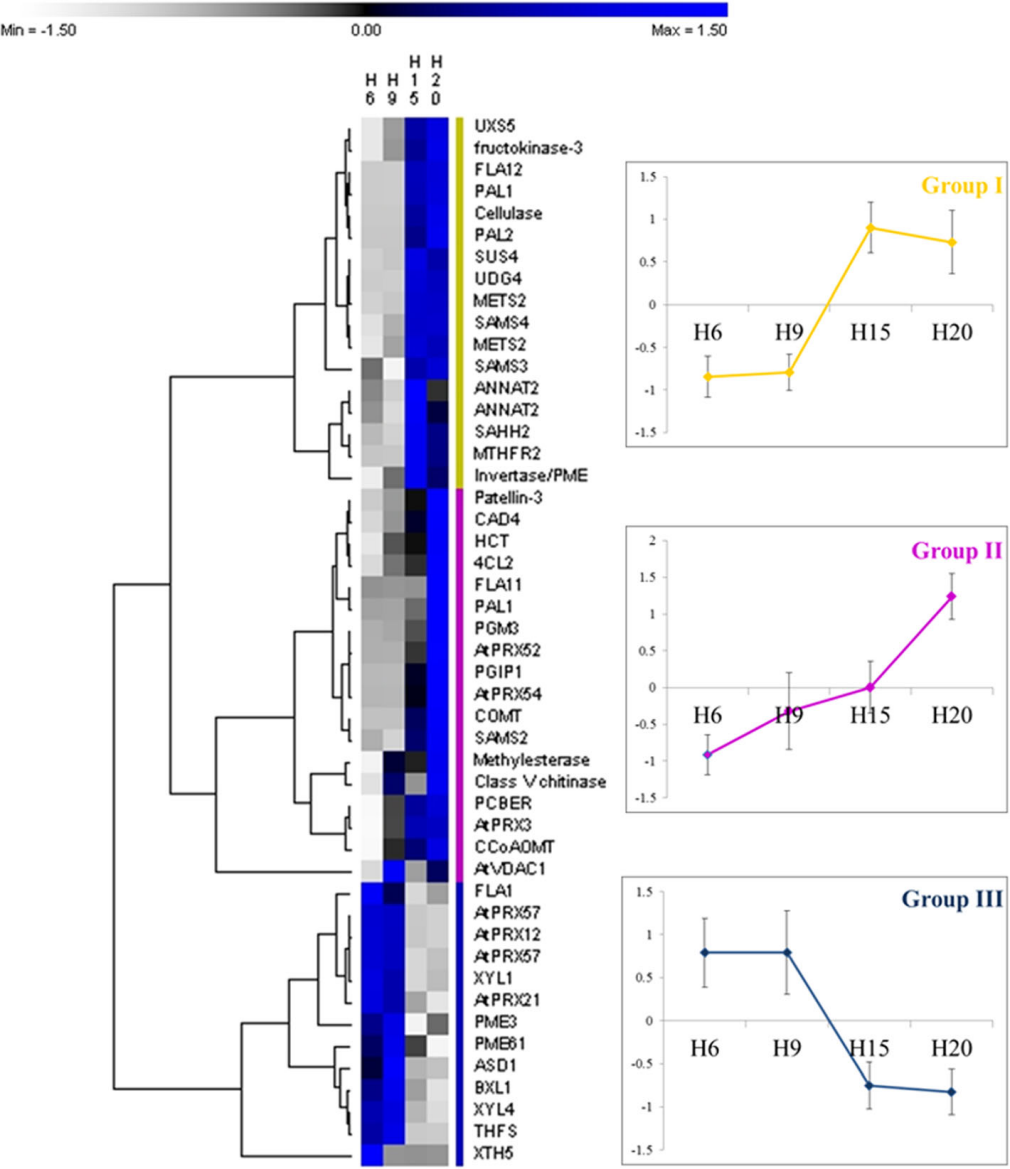
NSAF relative quantities of proteins involved in cell wall biogenesis assessed by LC-MS. The parameters of the hierarchical clustering are indicated in the Methods section. The values are given in Additional file 3. Abbreviations are as in the text. For each group, the average of the abundances as calculated for the hierarchical clustering was plotted (± standard deviation)

In the two-dimensional differential gel electrophoresis (2D–DiGE) experiment, 433 spots were reproducibly matched across the gels and used in a Principal Component Analysis (PCA, Fig. 5a). In the LC-MS experiment, only peptides with three or more spectral counts were considered for the Independent Component Analysis (ICA, Fig. 5b), resulting in 404 variables. Both approaches allowed to discriminate young (H6 and H9) from more mature (H15 and H20) hemp hypocotyls. Using LC-MS/MS, more proteins involved in cell wall formation and, more specifically, in monolignol biosynthesis, were identified and quantified. The patterns of abundance of these proteins are shown using the Normalized Spectral Abundance Factor (NSAF) value (Fig. 6).

When the proteins involved in cell wall development and monolignol synthesis are used for a hierarchical clustering (Fig. 6), three groups can be discerned. Group I (in yellow) includes proteins which are more abundant in H15 or H20 as compared to younger hypocotyls, namely UDP-XYLOSE SYNTHASE 5 (UXS5), FRUCTOKINASE-3 (FRK3), FASCICLIN-LIKE ARABINOGALACTAN 12 (FLA12), PAL1, CELLULASE, SUS4, UDP-GLUCOSE DEHYDROGENASE 4 (UDG4), METHIONINE SYNTHASE 2 (METS2), *S*-ADENOSYLMETHIONINE SYNTHASES 3–4 (SAMS3–4), *S*-ADENOSYL-L-HOMOCYSTEINE HYDROLASE 2 (SAHH2), METHYLENETETRAHYDROFOLATE REDUCTASE 2 (MTHFR2), ANNEXIN 2 and one invertase/PME. The patterns of gene expression of SAMS and METS (Fig. 4) are closely related to the abundance of their respective proteins. The proteins of the group II (in magenta) are more abundant in H20. They include the majority of the proteins involved in the phenylpropanoid and monolignol biosynthetic pathways, as well as in lignin polymerisation, such as PAL1, 4CL2, HCT, CAD4, COMT, CCoAOMT, PHENYLCOUMARAN BENZYLIC ETHER REDUCTASE (PCBER), and orthologs of AtPRX52, AtPRX54 and AtPRX3. Some proteins having a role in cell wall biosynthesis and modification are also present in this second group: PATELLIN-3, PHOSPHOGLUCOMUTASE 3 (PGM3), FLA11, POLYGALACTURONASE INHIBITING PROTEIN 1 (PGIP1), METHYLESTERASE, CLASS V CHITINASE, VOLTAGE DEPENDENT ANION CHANNEL 1 (VDAC1). The gene expression of other peroxidases involved in lignin polymerisation (orthologs of AtPRX49, AtPRX52 and AtPRX72) are similar to those detected in the LC-MS experiment, highlighting the ongoing lignification in the old hypocotyls. PATELLIN-3 is involved in the protein transport to the plasma membrane [27, 28]. VDAC1 is involved in the regulation of hydrogen peroxide generation [29] and thus may play a role in lignin polymerisation by the peroxidases. Finally, the proteins present in the group III (in blue) are more abundant in young hypocotyls. They are mainly devoted to the modification of the cell wall to ensure the extensibility of the hypocotyl: FLA1, PRXs, PMEs, XYLOGLUCAN ENDOTRANSGLUCOSYLASE/HYDROLASE 5 (XTH5), ALPHA-XYLOSDASE 1 (XYL1), BETA-XYLOSIDASE 1–4 (BXL1, XYIL4), and ALPHA-L-ARABINOFURANOSIDASE 1 (ASID1). 10-FORMYLTETRAHYDROFOLATE SYNTHETIASE (THFS) is involved in the metabolism of folic acid.

### Peroxidase activity

**Fig. 7:**
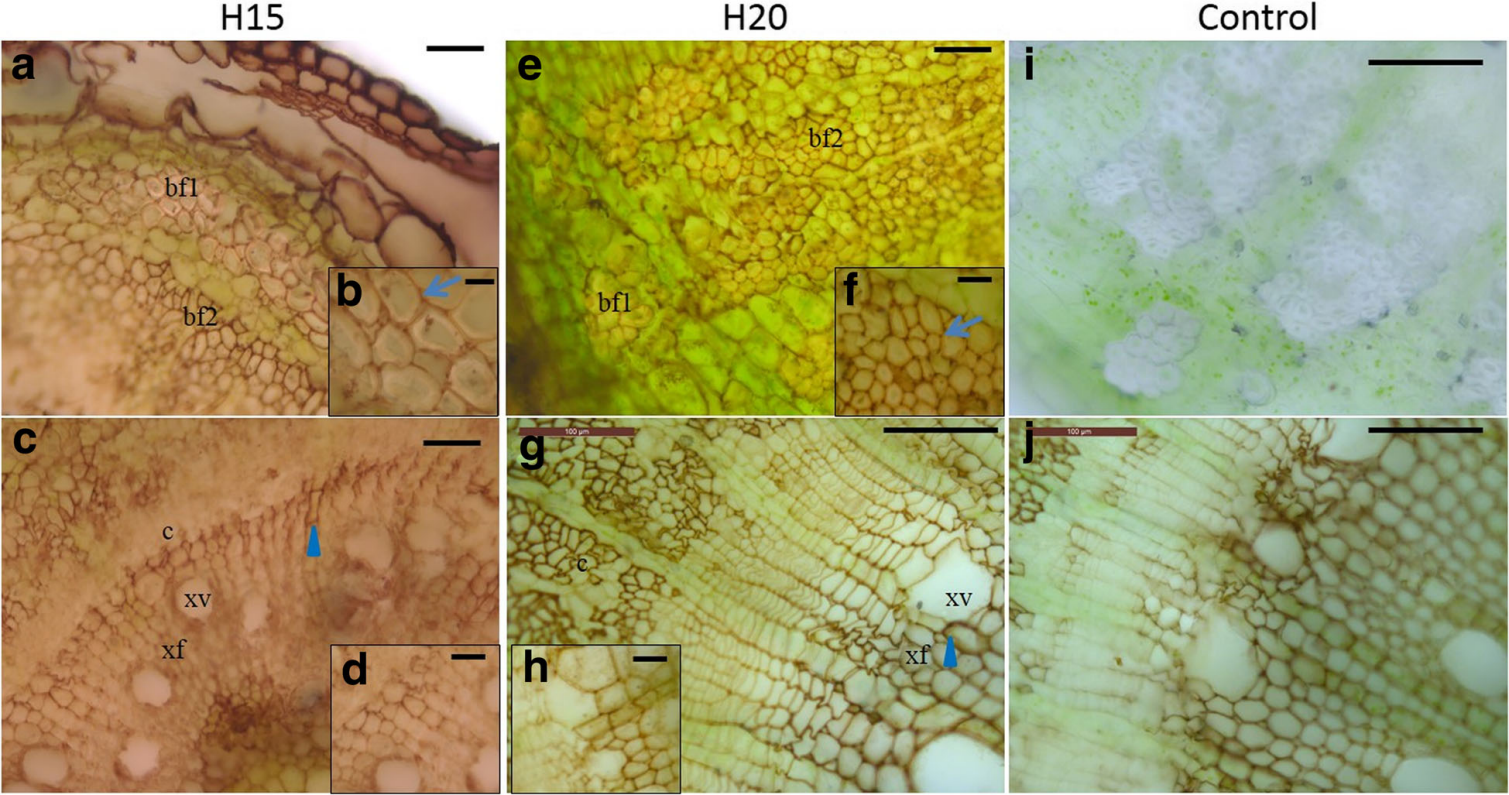
Peroxidase activity in H15 and H20. **a** to **d**, H15; E to H, H20. Details of the bast fibres and xylem regions are shown in **b** and **f** and **d** and **h**, respectively. Blue arrows indicate peroxidase activity in the middle lamella and cell corners of the bast fibres; blue arrowheads indicate peroxidase activity in the xylem vessels and fibres. **i**: negative control without DAB in H20. **j**: negative control with salicylhydroxamic acid as inhibitor of peroxidase activity in H20. bf1 primary bast fibre, bf2 secondary bast fibre, c cambial zone, xf xylem fibre, xv xylem vessel. Scale bar: 100 μm (**g**, **i**, **j**), 50 μm (**a**, **c**, **e**); 25 μm (**d**, **f**, **h**); 10 μ**m (b)**

The assessment of the peroxidase activity, as determined by the oxidation of 3,3′-diaminobenzidine, was done to complement the qualitative data related to lignin as visualised by the Mäule staining in H15 and H20. Overall, peroxidase activity was detected in the bast fibres, with a brown staining starting in the cell corners and extending to the middle lamella (Fig. 7a, b, e, f). In xylem cells, the activity was stronger in developing cells adjacent to the cambial region (Fig. 7c, d, g, h). The xylem fibres showed a more homogenous signal, from the cambium to the pith, while the vessels displayed a stronger staining in the young developing xylem (Fig. 7g). Application of salicylhydroxamic acid, an inhibitor of peroxidase activity [30], resulted in a decreased peroxidase activity in the cambial zone (Fig. 7j). This decrease was not so obvious in xylem and bast fibres, presumably because of the presence of secondary cell walls and lignin, hindering the penetration of salicylhydroxamic acid.

### Laccase activity

**Fig. 8:**
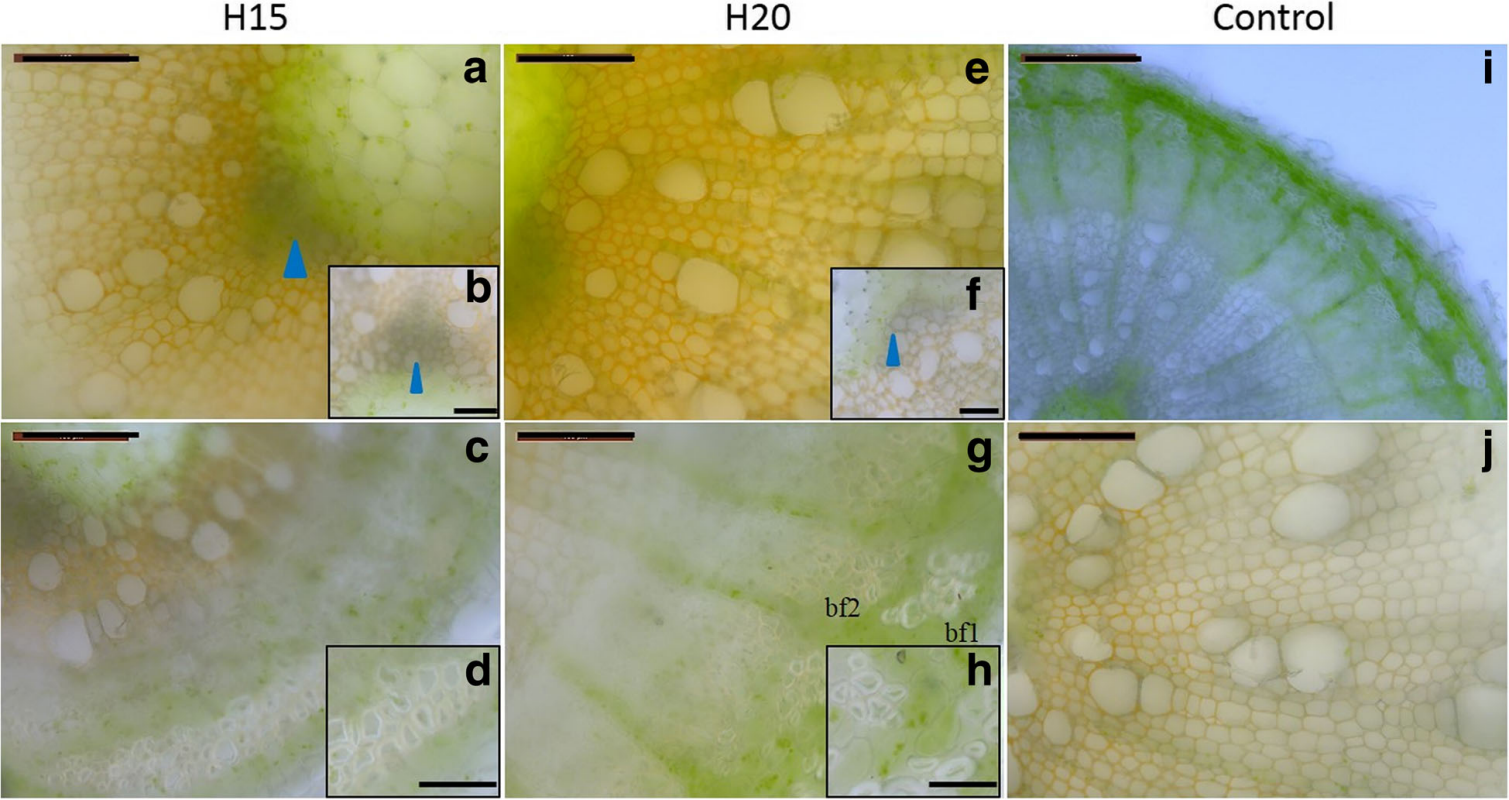
Laccase activity in H15 and H20. **a** to **d**, H15; **e** to **h**, H20. Details of the xylem regions and bast fibres are shown in **b** and **f** and **d** and **h**, respectively. Orange colour indicates the presence of laccase activity. Blue arrowheads indicate the absence of laccase activity in the lignified primary xylem. **i**: negative control without DAB in H20. **j**: negative control with sodium azide as inhibitor of laccase activity in H20. bf1 primary bast fibre, bf2 secondary bast fibre. Scale bar: 100 μm (50 μm in the insets)

A second activity stain, consisting in the oxidation of 3,3′-diaminobenzidine in the optimal pH range of laccase activity, was applied in hemp hypocotyls (Fig. 8). The signal was higher in the secondary xylem than in the bast fibres, both in H15 and H20 (Fig. 8c-g). We did not detect any staining in the primary xylem at any time point (Fig. 8a-b-f). In H15, the xylem vessels displayed a stronger staining with respect to the xylem fibres (Fig. 8c). In H20, the secondary bast fibres displayed a stronger laccase activity than primary fibres (Fig. 8g). Sodium azide was used as an inhibitor of laccase activity [31], and resulted in a decreased orange coloration (Fig. 8j).

## Discussion

The time-course study of the hemp hypocotyl aged between 6 and 20 days highlighted the roles and expression dynamics of several players involved in lignin and lignan biosynthesis. Two developmental stages, i.e. H6 and H9 versus H15 and H20, were discerned at the chemical (lignin accumulation and composition), protein (cell wall biogenesis and monolignol biosynthesis) and transcript levels (transcription factor related to secondary cell wall thickening, dirigent- and dirigent-like proteins, candidates involved in lignin polymerisation).

In this study, we have focused on two families of lignin-related genes, namely the *DIR* and *PLR*/*PRR*. In *Arabidopsis*, 25 DIR have been found. Since we have retrieved only 9 DIR genes in hemp, several other DIR-genes may be missing in our analysis. Two main groups of hemp DIR are clearly distinct from the phylogenetic analysis (Fig. 3). CsaDIR6A and CsaDIR6B belong to the same group as the *Arabidopsis*, *Schisandra chinensis*, *Forsythia* x *intermedia* and flax dirigent proteins (Group I). In those species, these DIRs are involved in the stereoselective initial coupling step of coniferyl alcohol to yield either (+)-pinoresinol or (−)-pinoresinol, a G(8–8)G lignan, in the presence of laccases [16]. We found that these hemp DIRs were more expressed at later stages of the hypocotyl development (H15 and H20) compared to H6 and H9 (Fig. 4). In *Arabidopsis*, PRR reduces (+)-pinoresinol to lariciresinol [32]. However, the patterns of expression of hemp *DIR6A*, *DIR6B*, *PLR* and *PRR1* did not overlap, since *PLR* and *PRR1* were more expressed in H6 and H9. It is therefore plausible that, in hemp, DIR6A and DIR6B may be involved in other biochemical reactions, such as those occurring in the ellagitannin or sesquiterpenoid pathways [33]. Various terpenes, including sesquiterpenes, have been detected in both medicinal and fibre varieties of *C. sativa* [34]. Several squalene epoxidases, key-enzymes of terpenoids biosyntheses, are expressed in the thale cress hypocotyl [35]. Therefore, the higher abundance of hemp *DIR6A* and *DIR6B* might be related to the biosynthesis of terpenes. Further analyses are required to determine the DIR involved in the stereoconformation of the lignans in hemp. Candidates may be found in the DIR-like proteins subfamilies, which regroup proteins whose functions are not yet clearly established [14]*.* The expression pattern of *CsaDLP4* is compatible with a role in lignan biosynthesis, as it is coexpressed with *PLR* and *PRR1*. The comparison with the co-expression data of genes related to *AtPRR1* may support this hypothesis. Indeed, the closest ortholog of CsaDLP4 (AtDIR18) according to the phylogenetic analysis (Fig. 3) is co-expressed with AtPRR1 (*p*-value <1E-03) (atted.jp). AtPRR1 is solely responsible for lignan biosynthesis in the stem [10]. However, in contrast with the expression of hemp *PRR1*, *AtPRR1* is co-expressed with many genes involved in secondary cell wall deposition such as *MYB46*, *SND1*, *CESA7* or *LAC17*. In a previous RNA-Seq-based study, the genes involved in secondary cell wall biosynthesis were more expressed either in H15 or in H20 [24]. It remains to be investigated whether the expression of *PLR* / *PRR* in our hemp system is transient and linked to the young developmental stage, or if it also applies to older phases.

Lignans positively (e.g., syringaresinol) or negatively (e.g., sesamin) regulate root and shoot lengths [4, 36]. A disruption in (+)-secoisolariciresinol diglucoside (SDG) biosynthesis by RNAi of flax *PLR* resulted in higher concentrations of dehydrodiconiferyl alcohol glucoside (DCG) and dihydro-dehydrodiconiferyl alcohol glucoside (DDCG) without compromising growth [37]. DCG promotes cell division, possibly by transducing the cytokinin signal [5]. This may indicate that the plant compensates the lack of SDG induced by the down-regulation of *PLR* by the biosynthesis of DCG. A detailed analysis of lignans present at the different time points of the hemp hypocotyl system may shed additional light on the exact functions of this class of molecules.

PLR expression and thus the synthesis and accumulation of lignans may also be involved in the response to oxidative stress. In *F. intermedia*, *PLR* mRNA is more abundant in young stems as compared to more mature stems and is localised in the vascular cambium and developing xylem [8]. During the early stages of xylem parenchyma cell development, the activity of PLR results in the synthesis of lignans, while lignification occurs in tracheary elements and fibres [8]. Xylem parenchyma cells, tracheary elements and fibres which did not yet complete their programmed cell death have to cope with oxidative intracellular conditions because of the production of H_2_O_2_ by xylem parenchyma cells [38]. H_2_O_2_ is able to diffuse from cell to cell, and may be used as a substrate for the activity of peroxidases involved in lignification. Niculaes and colleagues [39] propose that class I peroxidases catalyse the oxidation of monolignols and their subsequent dimerization into dilignols such as pinoresinol to prevent damages to the cells that have not completed their programmed cell death. The suggestion of a PLR protective function against oxidative stress in the developing xylem cells may explain its high expression in young hemp hypocotyls, besides its potential implication in the regulation of plant growth. Later in the development, the abundance of PCBER was higher (H15-H20, Fig. 6). In vitro, PCBER reduces dehydrodiconiferyl alcohol (DDC) to isodihydrodehydrodiconiferyl alcohol (IDDDC), a G(8–5)G neolignan. In living cells, such reduced products are oxidised by a peroxidase consuming H_2_O_2_, providing a protection against oxidative stress during lignification [39]. The authors suggest that PCBER and pinoresinol reductases prevent oxidative damage by producing radical scavenging molecules such as reduced phenylpropanoid coupling products. At the time points where PCBER was more abundant, one can speculate that neolignans are excreted to the secondary cell walls to cope with the oxidative stress accompanying lignin polymerization [9, 10]. Alternatively, PCBER may also be involved in xylem lignification by reducing the arylglycerol of S(8–5)G glycoside, whose product is finally used for scavenging H_2_O_2_ in the oxidative conditions found in lignifying tissues [39].

At the transcriptomic level, the ortholog of *Arabidopsis NST1*, a transcription factor involved in secondary cell wall deposition and lignification [7] was significantly more expressed in H15 and H20 as compared to H6 and H9 (Fig. 4). Consequently, most of the genes of the monolignol and lignin biosynthetic pathways were more expressed in older hypocotyls. The increased expression of *NST1* and genes of the monolignol-lignin biosynthesis is in accordance with the data retrieved in our previous RNA-Seq experiment [24]. During the proteome study, the abundance of enzymes involved in the generation of methyl donors was identified as significantly changing: METS, SAMS, SAHH and MTHFR were differentially abundant within the time course experiment (Fig. 6). The two proteins identified as the orthologs of AtMETS2 displayed different trends: the first was highly abundant at all the time points, with small but significant fold-change, while the second was following the same trend as the proteins of the monolignol pathway, with massive fold-change variations [Additional file 3]. This may point to different functions of these two isoforms. The same observation applies to the abundance of three isoforms of SAM synthases, the orthologs of AtSAMS2, AtSAMS3 and AtSAMS4, with SAMS2 and SAMS3 abundances remaining almost constant and SAMS4 being more abundant as the hypocotyl ages. Such changes in the abundance / expression profiles of the proteins / genes involved in the biosynthesis of methyl donors have also been documented in the flax hypocotyl, where genes of the *S*-adenosylmethionine pathway were more expressed at 15 days as compared to 6 days [40]. The methyl donors are involved in many biochemical reactions, including the methylation of G and S monolignol [41, 42].

The lignin content and composition depends on the activity of ca. ten enzymes, from PAL to COMT [43] (Fig. 1). Perturbations in the enzymatic activities of those enzymes lead to changes in lignin content, lignin composition, or both [44]. Accordingly, the higher abundances of PAL1, PAL2, 4CL2, CAD4, HCT, CCoAOMT and COMT in H15 and H20 correlate with the rise in lignin content (Fig. 6). The abundance pattern of the proteins involved in lignification is consistent with the transcriptomics data obtained with the RNA-Seq analysis of the same time points (Correlation plot in Additional file 3) [24]. The lignin composition also depends on the cell type (e.g., vessels versus fibres) and age (deposition and maturation of the secondary cell wall). H lignin accumulates in the middle lamella of vascular cells [16], prior to G and S lignin deposition in the S1 sublayer. H lignin is believed to be a factor determining the shape of the vascular cells [44]. Lignin rich in H units is more condensed because this subunit is capable of forming condensed units at the 3 and 5 positions [45]. The pectic nature of the middle lamella may explain why lignin is present in a more condensed substructure with a high H unit content [46, 47]. Indeed, the loose structure of the pectic matrix sterically favours the formation of the condensed lignin and accommodates its bulky organisation [48]. Noncellulosic polysaccharides such as galactan and xylan have roles in controlling the cellulose microfibril orientation, resulting in qualitative and quantitative changes in the lignification of the middle lamella and cell walls [47, 49]. In alfalfa, the middle lamella lignification occurs in specific spots where pectin, peroxidase activity and H2O2 are present [50].

During the hypocotyl elongation, the expression of several genes involved in lignification was shown to be under control of the circadian clock [51]. Among them, *C4H*, *COMT* and *CCoAOMT* show circadian-dependent expression in thale cress. More specifically, their transcripts are more abundant 4-8 h before dawn, when cell elongation is slowed down or has stopped. These results may be linked to the availability of metabolizable sugars for lignification [52]. In support of this hypothesis, these authors have shown that *sex1*, a mutant impaired in starch turnover resulting in a reduced pool of available carbon, accumulates less lignin than the wild type. In addition, the lignin of the *sex1* mutant was completely depleted of H unit because of the higher C3’H activity relative to other enzymes of the phenylpropanoid/monolignol pathway [52]. Moreover, sucrose supplied to dark-grown hypocotyl induces lignification [52]. Sucrose may be a signalling molecule to induce the activity of the lignin biosynthetic pathway. As a result, sucrose may be considered both as a source of carbon-rich skeletons for lignification, and as a signalling molecule regulating a suite of developmental processes, including the differentiation of xylem cells [52]. One may speculate that the deposition of H lignin in the elongating hypocotyl may be (partially) tuned by sugar availability. Several enzymes involved in sucrose, glucose and fructose metabolism (e.g. SUS4, INV, FRK3; Additional file 3 and Fig. 1) were more abundant in H15 and H20. Increased sucrose synthase activity leads to higher cellulose content, as it provides UDP-glucose, the precursor used by the cellulose synthase complex [53]. Invertase hydrolyses sucrose into glucose and fructose. Glc-6-P may be converted in erythrose-4-P to be shunted to the shikimate pathway, producing phenylalanine, a precursor for the biosynthesis of lignols (Fig. 1) [54]. Specifically in H20, most of the proteins associated with downstream phenylalanine metabolism, especially monolignol biosynthesis (PAL1, PAL2, 4CL2, CAD4, HCT, COMT and CCoAOMT) and lignin polymerisation (orthologs of AtPRX3, AtPRX52 and AtPRX54), reached their maximum abundance [Additional file 3]. Fructose is likely phosphorylated by FRK3 to avoid feedback inhibition of SUS4 and invertase, therefore contributing to cellulose biosynthesis. Fructose may also be converted to UDP-glucose and finally sucrose [55]*.*

As the hypocotyl ages, increased CCoAOMT and COMT abundances (Fig. 6) result in lignin richer in G and S subunits (Table 1). We therefore show a consistent link between proteomics data and lignin monomeric composition. The lignification of the secondary cell wall begins with deposition of G units in the S1 sub-layer [16] in discrete domains where LAC4 and LAC17 are present [20]. Despite the lack of a precise localisation of these two enzymes in the hemp hypocotyl system, we may however assume that LAC4 and LAC17 activities lead to a decrease in the relative proportion of H lignin (Table 1). This was previously observed in hemp bast fibres: the H lignin proportion of apical fibres was systematically higher than in basal fibres, irrespective of the stage of development [56]. Using thioacidolysis, the same authors found out that the S/G molar ratio was also higher in the basal fibres. Primary xylem is almost completely devoid of S lignin, as shown by the brown coloration of the Mäule staining (Fig. 2) and as previously described [57]. Lignin in the secondary xylem is progressively enriched in S units [57]. This rise in the S/G ratio was also observed during lignification of mature secondary cell walls in woody Angiosperms, mainly due to S lignin polymerisation [58]. The development of fibres in both xylem and phloem likewise contributes to this increase: fibres, as compared to tracheary elements, are richer in S lignin to provide mechanical strength. In *Quercus suber*, the xylem lignin is enriched in S units because of the large proportion of fibres (S/G of 1.2, [58]). The monomeric composition of lignin also depends on the carbohydrate composition of the cell wall where the polymerization occurs [46]. In secondary cell walls, elongated patches of lignin are deposited between the cellulose microfibrils, acting as a template to guide the lignification [48, 49]. Because secondary cell walls are richer in hemicellulose, e.g. xylan in the xylem cells of hemp [24, 59], than the middle lamella, lignification mainly occurs with polymerization of G and S subunits [46]. By controlling the orientation of the cellulose microfibrils in the secondary cell wall, xylan favours the formation of the microfibril matrix [48]. This, in turn, favours the formation of non-condensed lignin (mainly made of G and S subunits) and its extended conformation adapted to the tight volume available between the cellulose microfibrils [48]. For example, the *irx8* mutant, which is disturbed in the xylan architecture of the secondary cell wall, has less lignin because of the lower amount in G subunits [47]. In old cell walls, lignin and xylan are covalently linked to form the lignin-carbohydrate complex (LCC) by the addition of nucleophilic groups (hydroxyl or carboxylic groups of hemicelluloses) on the transient quinone methide intermediate, determining the final step of cell wall construction [60]. In hemp, primary and secondary phloems are rich in extraxylary fibres, which are already lignified in the hypocotyl aged 20 days (Fig. 2). However, the rise in the S/G ratio in the hypocotyl time course system differs from the trend observed in the outer tissues of adult plants, where the S/G ratio in the same stem fragment (apical or basal) does not change significantly when it gets older [56]. The authors suggest that the high amount of crystalline cellulose of the bast fibres may impair the polymerisation process. Since in our hypocotyl system the vascular tissue of the xylem is more developed than the sclerenchyma bast fibre tissue, we can observe an increase in the S/G ratio throughout time. Laser capture microdissection was found to be a reliable method to study the lignin composition of specific cell types in herbaceous species, where the manual separation of specific tissues is cumbersome [44] and may provide important data complementing the Mäule staining.

The synthesized monolignols are excreted to the cell wall where they are polymerised into lignin under the activities of laccases and class III peroxidases [47], with the possible intervention of DIR. The involvement of peroxidases in the lignification process is obvious when considering the abundances of both transcripts (orthologs of *AtPRX49*, *AtPRX52* and *AtPRX72*) and proteins (orthologs of AtPRX3, AtPRX52 and AtPRX54), which are in general higher at older stages of development (Figs. 4 and 6). The three transcripts were more abundant in H15, while the three proteins were more abundant in H20. Peroxidase activity is essential for fibre lignification in several species including *Arabidopsis* [21, 61] and flax [62] and is required for S lignin polymerisation, since laccases seem not to be able to catalyse this polymerisation [63]. Likewise, S lignin staining and peroxidase activity were overlapping in the bast fibres (Figs. 2 and 7). Moreover, the increase in the peroxidase activity observed during tracheary element differentiation (close to the cambial region) and lignification (Fig.7d, h) was previously described in *Zinnia* [63]. During the hemp hypocotyl development, secondary xylem cells are visible 12 days after sowing [24] and lignification is ongoing at least until day 20 (Table 1). The role of the two laccases LAC11 and LAC17 is more difficult to define, as there is no significant change in their expression when determined by RT-qPCR (Fig. 4). However, we have previously described the expression pattern of putative orthologs of *A. thaliana LAC17* using RNA-Seq in the same hypocotyl system, showing that one isoform of this gene was strongly upregulated in H20 [24]. As several isoforms of *LAC17* were found using RNA-Seq, it is plausible that the transcripts detected by the two methods are different isoforms. One may also speculate that there is a basal level of laccase activity until the hypocotyl reaches its final diameter, i.e. when all fibres and vessels have differentiated from the cambium and started their initial lignification with G units. Laccases are involved in secondary cell wall lignification of protoxylem tracheary elements in young elongating tissues [20]. In H15 and H20, no laccase activity signal was observed in the primary xylem (Fig. 8), but a strong peroxidase signal was instead visible (Fig. 7c). This may suggest that a peroxidase-driven lignification occurs after the polymerisation of monolignols by laccases. Berthet and colleagues [19] suggested that laccases are expressed at the beginning of the lignification and involved in the polymerisation of G-rich lignin in fibres. In flax, three orthologs of *AtLAC4* and *AtLAC17* were more abundant in the upper region of inner stem tissues, containing the xylem, while five peroxidases were more expressed in the lower region of inner stem tissues [9]. The proposed mechanism of lignification suggested by these two studies is compatible with the increase in the S/G ratio along the time course.

The role of the DIR in lignification is limited. Since lignin polymerisation is performed through end-wise radical coupling of phenols to the free-phenolic end of the growing polymer and not by enzymatic control [17], a direct role of DIR in this process is unlikely. However, they may play a role in lignin localisation in specific regions of the cell wall. By silencing *AtDIR10* (*ESB1*), perturbations in the organisation of Casparian strips have been observed [12]. The authors suggest that ESB1 plays a role in the localisation of lignin. In the phylogenetic analysis (Fig. 3), CsaDLP4 and CsaDLP5 fall into the same cluster as ESB1, possibly suggesting a role in lignification. The expression pattern of *DLP5* is in line with such a function, as it is more expressed in old hypocotyls and differently from *DLP4*. The hemp genes *DLP2*, *DLP20A* and *DLP20B* were more expressed in H15 and H20 (Fig. 4). These DLPs belong to another subgroup, from which no proteins have been functionally characterised yet. The need of such studies is obvious to understand the precise role of the DIR-like proteins.

## Conclusions

The molecular aspects of lignin and lignan biosyntheses in the hemp hypocotyl system were studied. Lignin content and composition were in line with proteomics, RT-qPCR and microscopic observations of laccase and peroxidase activities. These results foster our understanding of lignification during primary and secondary growth and open venues of functional studies of the mechanisms underlying primary and secondary cell wall lignification.

## Methods

### Plant growth and sampling

The hypocotyls have been grown and sampled following the conditions described in [24]. Each biological replicate consisted in 20 hypocotyls.

### Lignin analysis

#### Lignin quantification

Lignin content has been assessed on preparations of cell wall residue (CWR) of 4 or 5 biological replicates [62]. CWR was obtained by washing the powdered plant material first with methanol (80%) under agitation for 4 h, followed by five additional vortexing/centrifugation cycles with ethanol (80%). After drying, 5 mg of CWR were digested with 2.6 mL of 25% acetyl bromide in glacial acetic acid for 2 h at 50 °C using a Hach LT200 system. After digestion, the solution was transferred to a 50 mL Falcon tube containing 10 mL of 2 M sodium hydroxide and 12 mL of glacial acetic acid. The reaction tube was rinsed with glacial acetic acid and 1.75 mL of 0.5 M hydroxylammonium chloride was added. Finally, the total volume was adjusted to 30 mL with glacial acetic acid. The absorbance of the solution was read at 280 nm in a spectrophotometer, with an extinction coefficient of 22.9 g^−1^ L cm^−1^ for lignin determination.

#### Lignin characterisation

Lignin was characterised on 3 or 4 biological replicates using the nitrobenzene oxidation method [64]. 10 mg of CWR were digested with 2 mL of 2 M NaOH and 30 μL nitrobenzene at 165 °C for one hour (Hach LT200 system). After centrifugation, ca. 1500 μL of supernatant was collected and 10 μl of vanillin-D3 (Sigma-Aldrich) at 10 mg/mL in 1,4-dioxan were added as a surrogate standard. Nitrobenzene was removed by four washing steps with ethyl acetate (1 mL, vortexing/centrifugation cycle). The pH of the solution was adjusted to 2–3 by adding approximately 200 μL of 6 N HCl solution. The oxidation products were recovered by two successive extractions with 1 mL ethyl acetate (vortexing/centrifugation cycle) followed by cleaning with 500 μl of saturated NaCl solution and drying with Na_2_SO_4_. The GC-MS analysis was performed after trimethylsilylation, realized by addition of 50 μl of Bis(trimethylsilyl)trifluoroacetamide (BSTFA) to 50 μL of dried extract and derivatization at 60 °C for 30 min. Quantitative analyses were performed using a HP-5MS column (30 m × 0.25 mm, 0.25 μm, Agilent) installed in a 7890B-5977A GC-MS system (Agilent). Injection was done at 250 °C in splitless mode. The oven program started at 40 °C for 5 min, increased to 230 °C at 10 °C/min, then to 320 °C at 40 °C/min and was kept at 320 °C for 10 min. Salicylic acid-D4 was used as internal standard.

### Gene expression analysis

The gene expression analysis was carried out on biological triplicates using RT-qPCR. After grinding, RNA was extracted using the RNeasy Plant Mini Kit (Qiagen), treated with DNase I on column, and characterised with a NanoDrop 1000 Spectrophotometer (Thermo Scientific) and a 2100 Bioanalyzer (Agilent Life Sciences). The RNAs displayed a RIN value between 7.9 and 10. Reverse transcription was carried out with the ProtoScript II Reverse Transcriptase (NEB) following the manufacturer’s instructions. Primers were designed with Primer3 and validated for the absence of dimers and secondary structures (hairpin) using OligoAnalyzer 3.1 (http://eu.idtdna.com/calc/analyzer). qPCR runs were performed in 384 well-plates with the Takyon SYBR Green low ROX (Eurogentec), on a ViiA7 Real-Time PCR System (Applied Biosystems). The specificity of the products was checked at the end of each run with the melt curve. Relative gene expressions were determined using the qBase^PLUS^ software v2.5 (Biogazelle). *CsaETIF3e* and *CsaETIF3h* were the most stable reference genes among *ETIF3e, ETIF3h*, *Tubulin* and *ETIF4e*. The genes are named based on the putative orthology with the genes from *Arabidopsis*.

### Proteomics

#### Hemp hypocotyl soluble protein extraction

The proteomics experiments were carried out with five biological replicates using both gel-based and gel-free methods. Approximately 300 mg of material were treated with ice-cold extraction buffer (TCA 20%, DTT 0.1% in acetone) and allowed to precipitate overnight at −20 °C. After centrifugation (30,000 g; 45 min at 4 °C), the pellet was washed three times in ice-cold acetone and dried in vacuo. The dried extract was solubilised in 500 μl labelling buffer (7 M urea, 2 M thiourea, 2% CHAPS, 30 mM Tris) for 30 mg and incubated at room temperature under agitation (900 rpm) for 1 h. After centrifugation (15,000 g, 15 min), the supernatant was transferred to a fresh tube and pH was adjusted at 8.5 with sodium hydroxide (50 mM). Protein concentration was determined using the 2-D Quant Kit (GE Healthcare) with BSA for the standard curve according to the protocol defined by the manufacturer.

#### Gel-based proteome study

50 μg of protein were labelled with Cy-dyes. Following the labelling, the samples were handled as described in [65]. The analysis of the gel images was performed with the DeCyder™ software (GE Healthcare, v. 7.0.8.53). Spots were considered as significantly different when detected on at least 75% of analysed gel images, protein abundance with a minimum fold change of 1.5 with a Student’s t-test *p*-value below 0.05 [Additional file 3]. Following MALDI analysis, the mass spectra of digested peptides were identified by carrying out a MASCOT database search against our in-house hemp transcriptome database (170,598 sequences; 64,508,806 residues) annotated using Blast2GO PRO version 3.0 against the *A. thaliana* non-redundant database and the NCBI Viridiplantae database, with the following parameters: fixed modifications: carbamidomethyl (C); variable modifications: dioxidation (W), oxidation (HW), oxidation (M), Trp → kynurenin (W); mass values: monoisotopic; peptide mass tolerance: ± 100 ppm; fragment mass tolerance: ± 0.5 Da and Max number of missed cleavages: 2. Individual ions scores greater than 42 indicate identity or extensive homology (*p* < 0.05), protein scores greater than 65 are significant (p < 0.05). A protein was identified with only one peptide if the individual ion score was higher than 84. Principal Component Analysis (PCA) was performed with the DeCyder™ software.

#### Gel-free proteome study

25 μg of proteins were loaded in a Criterion™ XT precast 1D gel 4–12% Bis-Tris (1.0 mm X 12 wells, Bio-Rad). After denaturation of the sample, migration was performed at 200 V during 8 min. The gel was stained with Instant Blue (Gentaur BVBA) for 1 h. Bands were excised and cut into 1–2 mm cubes at 4 °C. The proteins in each band were subsequently reduced (NH_4_HCO_3_ 100 mM + DTT 10 mM, 30 min at 56 °C), alkylated (NH_4_HCO_3_ 100 mM + IAA 55 mM, 20 min at room temperature), de-stained and digested with trypsin (5 ng/μl in NH_4_HCO_3_ 50 mM, 30 min on ice and 37 °C overnight). Peptides were extracted from the gel with ACN 50% / TFA 0.1%, dried with a Speedvac and stored at −20 °C until LC-MS analysis. Peptides were analysed with a NanoLC-2D System (Eksigent) coupled to a TripleTOF 5600+ MS (AB Sciex). After desalting and enrichment (C18 PepMap™, 5 μm, 5 mm * 300 μm, Thermo scientific), the peptides were separated with a C18 reverse phase column (PepMap™ 100, 3 μm, 100 Å, 75 μm × 15 cm, Thermo scientific) using a linear binary gradient (solvent A: 0.1% formic acid; solvent B: 80% ACN, 0.1% formic acid) at a flow rate of 300 nl/min. Peptides were eluted from 5% to 55% solvent B over 40 min, afterwards eluent B increased to 100% to wash the column and the column was re-equilibrated. The LC was coupled to the mass spectrometer with a NanoSpray III source. CID fragmentations for MS/MS spectra acquisitions used the automatically adjusted system of rolling collision energy voltage. A full MS scan was performed (scan range: 300–1250 m/z, accumulation time: 250 ms) and the 20 most intense precursors selected for fragmentation. The CID spectra was analysed with Mascot-Daemon using the hemp database as for gel-based proteomics using the following parameters: 2 missed cleavages, mass accuracy precursor: 20 ppm, mass accuracy fragments: ± 0.3 Da, fixed modifications: carbamidomethyl (C), dynamic modifications: Oxidation (M), Acetyl (protein N-term). Only the contigs where at least one time point has 3 (out of 5) or more than 1 spectral count were considered for further analysis. This filter being applied, a value of 0.5 was added to all the spectral count to compensate for null values and allowing logarithmic transformation [66]. The relative quantities of the proteins have been calculated using the NSAF.

NSAF = (100*SpC/MW)/Σ(SpC/MW)N, where SpC = Spectral Counts, MW = Protein molecular weight in Da and N = Total Number of Proteins [67].

The Independent Component Analysis (ICA) has been calculated with MetaGeneAlyse [68] using the NSAF values. Student’s T-tests on the fold-change between the time-points have been calculated with ln-transformed values based on the five biological replicates [Additional file 3].

Finally, the list of the differentially abundant proteins was obtained using the same parameters as for 2D–DiGE (fold-change (NSAF) > 1.5 and Student’s t-test *p*-value below 0.05). The NSAF values have been displayed using PermutMatrix [69] with the following parameters: dissimilarity assessed by Pearson distance, clustering in complete linkage, seriation and tree seriation in multiple-fragment heuristic (MF), rows normalized by Z-score scaling. The proteins are named following the *Arabidopsis* nomenclature.

### Imaging

#### FASGA and Mäule staining

For FASGA imaging, hemp hypocotyls were embedded in resin as described in [24], and cut at a thickness of 10 μm with a microtome. Cross sections deposited on microscopic slides were incubated for 15 min in the FASGA solution at 55 °C, rinsed with pure water and observed with an optical microscope (Leica DMR). Mäule staining was performed on fresh hand-cut sections. Sections were incubated in permanganate solution at 1% (*w*/*v*) for 5 min, rinsed with pure water, washed with 3.6% hydrochloric acid, mounted in saturated ammonia solution and immediately observed with an optical microscope (Leica DMR).

#### Peroxidase and laccase activities

Fresh hand cross-sections of H15 and H20 were treated with metal enhanced 3,3′-diaminobenzidine (DAB) substrate kit (ThermoFisher, number 34065), which reacts with horseradish peroxidase in presence of peroxide. Before the incubation for laccase activity, sections were incubated with catalase (100 μg.mL^−1^ in TBS pH 7) for 3 h at room temperature. The DAB solution was diluted 10-fold in stable peroxide buffer [70] for peroxidase activity and in TBS (pH 6) containing 100 μg.mL^−1^ of catalase for laccase activity. Sections were incubated at room temperature for 15 to 30 min, rinsed twice with water, mounted in water and immediately observed with an optical microscope (Leica DMR). A negative control without DAB solution was included for each time-point, resulting in the absence of the brown signal for both laccase and peroxidase assays. Salicylhydroxamic acid (5 mM) was used as an inhibitor of peroxidase activity [30] and sodium azide (1 mM) as an inhibitor of laccase activity [31].

## Additional files

Additional file 1: provides the protein sequences used for the phylogenetic analysis (Fig. 3). (PDF 22 kb)

Additional file 2: provides the CNRQ from the RT-qPCR experiment used for the Fig. 4. (XLS 13 kb)

Additional file 3: provides the proteomics data from gel-based and gel-free experiments. (XLS 116 kb)

## Abbreviations

2D-DiGE: two-dimensional differential gel electrophoresis
4CL: 4-coumarate ligase
ASD1: Alpha-l-arabinofuranosidase 1
BXL1/XYL4: Beta-xylosidase 1–4
C3’H: Coumarate 3-hydroxylase
C4H: Cinnamate-4-hydroxylase
CAD: Cinnamyl alcohol dehydrogenase
CCoAOMT: Caffeoyl-CoA 3-O-methyltransferase
CCR: Cinnamoyl CoA reductase
CNRQ: Calibrated normalized relative quantities
COMT: Caffeate O-methyltransferase
CWR: Cell wall residue
DDC: Dehydrodiconiferyl alcohol
DIR: Dirigent protein
DLP: Dirigent-like protein
F5H: Ferulate 5-hydroxylase
FLA: Fasciclin-like arabinogalactan protein
FRK: Fructokinase
G: Guaiacyl
H: *p*-coumaryl
HCT: Hydroxycinnamoyl transferase
IDDDC: Isodihydrodehydrodiconiferyl alcohol
INV: Invertase
LAC: Laccase
METS2: Methionine synthase 2
MTHFR2: Methylenetetrahydrofolate reductase 2
NBO: Nitrobenzene oxidation
NSAF: Normalized spectral abundance factor
PAL: Phenylalanine ammonia lyase
PCBER: Phenylcoumaran benzylic ether reductase
PGIP1: Polygalacturonase inhibiting protein 1
PGM3: Phosphoglucomutase 3
PLR: Pinoresinol lariciresinol reductase
PRR: Pinoresinol reductase
PRX: Peroxidase
S: Syringyl
SAHH2: *S*-adenosyl-l-homocysteine hydrolase 2
SAMS: *S*-adenosylmethionine synthase
SUS4: Sucrose synthase 4
THFS: 10-formyltetrahydrofolate synthetase
UDG4: UDP-glucose dehydrogenase 4
UXS5: UDP-xylose synthase 5
VDAC1: Voltage dependent anion channel 1
XTH5: Xyloglucan endotransglucosylase/hydrolase 5
XYL1: Alpha-xylosidase 1
