# Supplementary material for: Insights into the molecular regulation of monolignol-derived product biosynthesis in the growing hemp hypocotyl
Source: BMC Plant Biol. 2018 Jan 2;18:1. doi: 10.1186/s12870-017-1213-1 (PMC5749015; doi:10.1186/s12870-017-1213-1)
Supplement: Supplementary file 1 — provides the protein sequences used for the phylogenetic analysis (Fig. 3). (PDF 22 kb) [file 12870_2017_1213_MOESM1_ESM.pdf]

Insights Into The Molecular Regulation Of Monolignol-derived Product Biosynthesis In The Growing Hemp Hypocotyl

Marc Behr, Kjell Sergeant, Céline Leclercq, Sébastien Planchon, Cédric Guignard, Audrey Lenouvel, Jenny Renaut, Jean-Francois Hausman, Stanley Lutts, Gea Guerriero.

Additional file 1: Protein sequences used for the phylogenetic analysis

>AtDIR1-At5g42510

MAKRFLLLLPLLSSILLLAVSVTAYSTTTPYQGYKPEKFTHLHFYFHDVISGDKPTAVKVAEA
RPTTTLNVKFGVIMIADDPLTEGPDPSSKEVGRAQGMYASTAMKDIVFTMVFNYVFTAGEF
NGSTIAVYGRNDIFSKVRELPIIGGTGAFRFARGYALPKTYKIVGLDAVVEYNVFIWH

>AtDIR2-At5g42500

MAKRFLLLLPLLSTILLLSVSVTESEAYSTTKPCQGYKPDKFTHLHFYFHDVISGDKPTAVKV
AEARRTNSSNVNFGVIMIADDPLTEGPDPSSKEVGRAQGMYALTAMKNISFTMVFNLAFTA
GEFNGSTVAMYGRNEIFSKVREMPIIGGTGAFRFARGYAQAKTYKVVGLDAVVEYNVFIWH

>AtDIR5-At1g64160

MVGQMKSFLFLFVFLVLTKTVISARKPSKSQPKPCKNFVLYYHDIMFGVDDVQNATSAAVTN
PPGLGNFKFGKLVIFDDPMTIDKNFQSEPVARAQGFYFYDMKNDYNAWFAYTLVFNSTQHK
GTLNIMGADLMMVQSRDLSVVGGTGDFFMSRGIVTFETDTFEGAKYFRVKMDIKLYECY

>AtDIR6-AT4G23690

MAFLVEKQLFKALFSFFLLVLLFSDTVLSFRKTIDQKKPCKHFSFYFHDILYDGDNVANATSA
AIVSPPGLGNFKFGKFVIFDGPITMDKNYLSKPVARAQGFYFYDMKMDFNSWFSYTLVFNS
TEHKGTLNIMGADLMMEPTRDLSVVGGTGDFFMARGIATFVTDLFQGAKYFRVKMDIKLYE
CY

>AtDIR9-At2g39430

MAKALHITIFLFLISSNLLAFINSARLLDEIQPQPQLVPTGQIPTVAPTEAEEEDGTDDNPGLAT
TTTTASAVTVPAGPAEATEPLLEFFMHDVLGGSHPSARVVTGIVAQTEVNGIPFSKASNSIFP
VDNGVPLVNSNNINSVINPNTAPLLTGLGGAQTSTVIQNTNGNSNDALSANSLPFVTAGNLP
PGAALQHLMFGTITVVDDELTESHELGSAVIGRAQGFYLASSLDGTSQTLSLTVLLHGEHDQ
HDTLDDAISFFGVHRTASHASQIAVIGGTGKFEHAKGYAIVETLHNQDNQHITDGQDTILHFS
VYLTYKA

>AtDIR10-At2g28670

MAGQKILSLLVIALVVTFAAAARLLDEENAFSATTTTLGSGSGSTGIGFGAGTGSSGSGSTG
FGFGAGSGSSGSGSTGSGLGAGTGSIPSSGSGPGLLPTASSVPGSLAGGGSGSLPTTGSA
TGAGAGTGSALGGGPGAGSALGGGAGAGPALGGGAGAGPALGGGAGAGSALGGGGAG
AGPALGGGGAGAGPALGGGVAGSGSALGGGASAGPDNTLVFFMHDILGGSNPTARAVTG
VVANPALSGQLPFAKPNGANLPVSNGVPSNNNNNGIVNNNNVPFLVGLGGTTANILQNNNN
GNNILNGFPVASGGQLPSGSALQMLMFGTMTVIDDELTEGHELGSGLLGKAQGYYVASAID
GTSQTMAFTAMFESGGYEDSISFFGVLRTAVSESHIGVMGGTGKYVNARGFAILKTFTGSS
GTQQNQPHQFTDGLETVVECTVYLSY

>AtDIR11-At1g22900

MATPFLLLLLPLIFSTVLLLTITVTQSKPYSKTTPFQGNKPDKLTHLHFYFHDIISGDKPTTIRV
AEAPGTNSSATVFGAVLIVDAPVTEGPELSSKEVGRAQGLYASTDMKTFGFTMVFNFVFTE
GEFNGSTAALYGRNPILLEERELPIIGGTGDFRFARGYALPKTYKVVNIDAVVEYNVFIWH

>AtDIR12-At4g11180

MTNQIYKQVFSFFLSVLLLQSSTVSYVPKSFDLKKPCKHFVLYLHNIAYDGDNAANATAATIV
KPLGLGDHSFGELIIINNPVTLDQNYLSKPVARAQGFYFYNMKTNYNAWVAWTLVFNSTKH
KGTFTIMDANPFGLQPARDLSIVGGTGDFLMTRGIATFKTKLTQGSKYFCVEMNIKLYECY

>AtDIR13-At4g11190

MANQIYIISLIFLSVLLYQSTTVLSFRQPFNLAKPCKRFVFYLHNVAYDGDNTDNATSAAIVNP
LGLGDFSFGKFVIMDNPVTMDQNMLSEQVARVQGFFFYHGKTKYDTWLSWSVVFNSTQH
KGALNIMGENAFMEPTRDLPVVGGTGDFVMTRGIATFMTDLVEGSKYFRVKMDIKLYECYY

>AtDIR14-At4g11210

MANQIYLFSLICLSVLLCQSYTVSSFQKSLDLAKPCKRFVLHLHDIAYDGDNAANATSAAIVN
PLGLGDFSFGKFVIMDDPVTMDQNYLSKPVARVQGFFCYHGKATYDAWIAWTVVFNSTQH
KGAFTIMGENPFMEPTRDLPIVGGTGDFIMTRGIATLTTDHIDGSKYFRVKLDIKLYECYH

>AtDIR16-At3g24020

MMIKQSPFLLLTTILFTVAVFVAALDPAPEDPIFELYMHDLLGGSSPTARPITGLLGNIYNGQV
PFAKQIGFTPPENGIAIPNANGALPTVNGINGVPLGTGLSGTAYSGQNLNGIQTQLGPDGLS
LGFGTITVIDDILTSGPDLGSQPLGKAQGVYVASSADGSTQMMAFTAMLEGGEYNDNLNFY
GIYRIGSAMSHLSVTGGTGRFKNACGFAEVRPLIPSGQHEVDGAESLLRIIVHLKY

>AtDIR17-CAB67637

MEDTGSIKQEAQSHPPGIFEIPGEPAVVINGVPDEPQTDCMIAKDEPISSGTVGSGEWLEGR
EVRKFFLGRYYSGTVTKFDKQSGWYRVEYEDGDSEDLDWSELEEVLLPLDTKNSNTNAQS
EYGEAGQRVNVKAPYPGHKPEKLVTTTVKAPYPGHKPEKLIPLVDDILTVGPEITSEEVGRA
QGIFASADQNNFGLLMAFNFVFTKGEFSGSTVSMYGRNPIFSKVREMPIIGGTGAFRFGRG
YAQAKTFTFNTTSGNAVVKYNVYIWH

>AtDIR18-At4g13580

MMKQSPFSLLTSIFLIAALFTATTALDPAPEDPIFELYMHDILGGSSPTARPITGLLGNIYNGQ
VPFAKQIGFVPPQNGVAIPNANGAMPTVNGINGIPLGTGLSGTAFSGQNLNGIQTQLGPDGL
SLGFGTITVIDDIITSGPDLGSQPLGKAQGVYVASSADGSTQMMAFTAMLEGGEYNDNLNF
YGIYRIGSAMSHLSVTGGTGRFKNACGFAEVRPLIPAGQHFVDGAEMLLRIIVHLKY

>AtDIR21-At1g65870

MASLYLLLLLPLFLALILAATITESKSFSTTVKAPYPGHKPDKLTHLHFYFHDIVSGDKPTSVQ
VANGPTTNSSATGFGLVAVVDDKLTVGPEITSEEVGRAQGMYASADQNKLGLLMAFNLVFT
KGKFSDSTVAMYGRNPVLSKVREMPIIGGTGAFRFGRGYALAKTLVFNITSGDAVVEYNVYI
WH

>AtDIR23-At2g21100

MAKEEYVSRMLVMLIMIMPLVAQGSRLHSWANRLEETGKDKVTNLQFYFHDTLSGKNPTAV
KVAQGTDTEKSPTLFGAVFMVDDALTETADPKSKLVGRAQGLYGSSCKEEVGLIMAMSFCF
EDGPYKDSTISMIGKNSAMNPIREMPIVGGTGMFRMARGYAIARTNWFDPKTGDAIVGYNV
TIMH

>AtDIR24-At3g55230

MAKALSLTIFLFLLIASNVQSARLLDEVQTQPQLVPQVPEEEDDSPQAVTTTPTPIPLPGPAT
GGPEPILEFFMHDVLGGSHPSARVVTGIVAQTEVNGIPFSKSSNNIFPVDNAVPLVNANSIN
NLINPNTAPLLTGLSGSQANTVIQNSNGNSQGSLSSNNLPFVTTGQLPPIAALQQLMFGSITV
VDDELTEGHELGSAIIGRAQGFYLASSLDGTSQTLSLTVLLHEDHDHHDTLDDAISFFGVHR
TASHASHIAVVGGTGRFEHAKGYAVVETLHNQEDQHVTDGHDTILHFSVYLTYYKA

To retrieve the dirigent proteins from Cannabis sativa, a consensus sequence of 20 DIR/DLP from Arabidopsis thaliana was built and a blat analysis was performed against the C. sativa genome (van Bakel et al., 2011) in the database from the University of Toronto (http://genome.ccbr.utoronto.ca/cgibin/hgBlat?command=start&org=C.+sativa&db=finola1&hgsid=73962). The output results were then processed with the blastx algorithm from the MPGR resource (http://medicinalplantgenomics.msu.edu/mpgr_blast.shtml) and the resulting sequences were used for designing the primers for RT-qPCR.

>Csa-DIR6A -csa_locus_13101_iso_4_len_657_ver_2

MMRGDHTSQKLAFSFILLLIIVLASSQSALASKKPLNEKSPCKRFVLYYHDTLFNGTDAANAT
SATVANKTRLGDFNFGMLVVFDDPITKDNHLLSPPVARAQGFFFYDKKNEYNAWFAFTLVF
NSSEYKGTLNIMGADLMPEKTRDLSVVGGTGDFFMARGIVTIETDTLQGDFYFRLKMDIKLY
ECY

>CsaDIR6B- csa_locus_18252_iso_1_len_450_ver_2

MKSPKPIFLSLILFFIIIFINDFNNALPQSSSMMKNNKLIKPCKSLVFYFHDIIYNGENAKNATSA
IVGAPAWGNKTLLAGKNHFGDLVVFDDPITLDNNLHSAPVGRAQGFYLYDKKDVFTAWLGF
SFVFNSTEHKGSLNFAGADPLMNKTRDVSVVGGTGDFFMSRGIATLMTDAFEGEVYFRLR
VHIKLYECWL

>Csa-DLP1-csa_locus_63472_iso_1_len_673_ver_2

MAKALQTTTLNLLISLTILLSFLRLTTANDDYYNSVVQRKHGFKHEKLTHFHFYFHDTVSGKN
PTNVQVAEAPTTNTSATFFGMVAVLDDPLTVGPEPTSKQVGRAQGIIAMASLSDVGLLMSL
NYVFTEGKYNGSCLSILGHNSILSALREMPVVGGTGLFRFARGYALAKTYMFNATSHDAIVE
YNVYVLHF

>CsaDLP2-csa_locus_13020_iso_3_len_979_ver_2

MEEALISKTQLTILSFLLTLTLVFALGKAEDGFVRAMDRKLLGLKKQKFSHFRFYWHDIYSGK
NPSALPIIQPPKNSSKNGFGVVSMIDDPLTEGPELSSKLLGKAQGFYGLASQEEIALIMAMNF
HIMQGKYNGSTLTILGRNNVFNKVREMPVIGGSGLFRFASGYAHASTHKFNPSNGDAVVEY
NVYVQHY

>CsaDLP3-EST01417

MKNSSTGFGFVVMMDDPLTVGPELSSKQVGRAQGMYGSASQSEWGLLMVLNYVFTEGK
YNGSTLSILGRNAVFSEVREMPIVGGSGLFRFARGYAQAKTHKFDLKTGNAVVEYNVYVLH
Y

>Csa-DLP4-csa_locus_19404_iso_1_len_1338_ver_2

MLKSQSVITLLFLTLISVYNPILLVAAVVPVGEGEPVLEMYMHDILGGSNPTARPVTGLLGNIY
SSQVPFAKPIGFKTPDDAVALPNANGAMPTVNGVTGLPLGTGLAGTAFAGNVNNQNTNNL
QSQLAADGLGLGFGTITVIDDILTTTPELGSQQLGKAQGVYVASSADGSKQLMAFTALMEG

GEFNDNLNFFGVYTIGTTLSQLSVTGGTGKFKNAYGIAELRPLIPPGQISTDGAETLLRITVHLKY

>CsaDLP5-csa_locus_61511_iso_1_len_851_ver_2

MRKDMSKLSCITLACMILLLIFNQSSSTRTLGNSVPNPTRHNYNHNHHKITFLMRDIFNVTTSTKLTSTTTNDLPFSKPLGLIPPKDGVRVPSTESFPQMLSFPGLSFPMARATLQELEFGTVTPIEEGIYNSDHSHHHDDSLRVVIGKAQGIYVATSEGGISSHMMALTASFGDGDEANGLRFFGVRKKGVTESHIAVIGGVGKYQGANGYATLKRVNINSQKNNGVSKLFKFSVYL

>CsaDLP20A-csa_locus_62520_iso_1_len_631_ver_2

MVIPPQANYSSTSFGMVAMIDDPLTEGPELSSKIVGKAQGFYGSASQEEVGLIMAMNFAFSEGKYNGSTITILGRNTVFSKVREMPVIGGSGMFRFARGYVQARTHQFTPSSGDAIVEYNVFVLHY

>CsaDLP20B-csa_locus_33311_iso_1_len_752_ver_2

MDKLTIQRITPTFLFLFIFIFIVKTLATATKSDYEFDHKGQIFSRNLSRKKLGLHKAEKVSHLHFYFHDVVVGRNPTTIVVAEGKAMKNSSTGFGFVVMMDDPLTVGPELSSKQVGRAQGMYGSASQSEWGLLMVLNYVFTEGKYNGSTLSILGRNAVFSEVREMPIVGGSGLFRFARGYAQAKTHKFDLKTGNAVVEYNVYVLHY

>LuDIR1

MAISRSNIALFFIFFICLSSANSSAKKKQHTPCKELVLFFHDIIYNGHNKANATAAIVAAPEGANRTILAGEFHFGNIAVFDDPITLDNNLHSPPVGRAQGMYLYDTKNTFTAWLGFTFSLNSTEHQGTINFMGADPLMNKTRDVSIVGGTGDFFMHRGVATIMTDSYEGEVYFRLRVDMKFYDCW

>LuDIR2

MTISRSKIALFFIFFIYLSSTPSSAKKKQHAPCKELVLFFHDIIYNGQNKANATAAIVAAPEGSNRTILAGESHFGNIAVFDDPITLDNNLHSPPVGRAQGMYLYDTKNTFTAWLGFTFCINSTEHQGTINFMGADPLMNKTRDVSIVGGTGDFFMHRGVATIMTDSYEGDVYFRLRVDMKFYECW

>LuDIR3

MTISRSKIALFFIFFIYLSSTSSSAKKKQHAPCKELVLFFHDIIYNGQNKANATAAIVAAPEGSNRTILAGESHFGNIAVFDDPITLDNNLHSPPVGRAQGMYLYDTKNTFTAWLGFTFCLNSTEHQGTINFMGADPLMNKTRDVSIVGGTGDFFMHRGVATIMTDSYEGEVYFRLRVDMKFYDCW

>LuDIR5

MKHSSSHSSSCLPFLLTTTTPIFLLLLSLICPAAATWRTPTHHQHGRNPNKPCKQLVLYYHDILFHGNGDQGNATSAAAANATKLGDYKFGMLVVFDDPVTKDGHLKSKAVARAQGFYFYDMKSTYNAWFAYTLVFNSTEHKGTINIMGADMMSEKTRDLSVVGGTGDFFMARGIATFRTDTFQGDNYFRLEMDIKLYDCYKY

>LuDIR6

MKHTSSFHFLLTTTTLIFLLLLSLISPGDATWRTPSHHLQHAKHPKPCKQLVLYYHDILFHGNGDQGNATSAAAANATKLGDYKFGMLVVFDDPVTKDGHLKSKAVARAQGFYFYDMKSTYNAWFAYTLVFNSTDHKGTLNIMGADMMSEETRDLSVVGGTGDFFMARGIATFRTDTFQGDAYFRLEMDIKLYECY

>Forsythia x intermedia psd_Fi1

MVSKTQIVALFLCFLTSTSSATYGRKPRPRRPCKELVFYFHDVLFKGNNYHNATSAIVGSPQ
WGNKTAMAVPFNYGDLVVFDDPITLDNNLHSPPVGRAQGMYFYDQKNTYNAWLGFSFLFN
STKYVGTLNFAGADPLLNKTRDISVIGGTGDFFMARGVATLMTDAFEGDVYFRLRVDINLYE
CW

>Schisandra chinensis DP

MEGRKLIITIPLLLFFIAFFSVPPAAFGRKVTLPRKRMPQPCMNLVFYFHDILYNGKNAANATS
AIVGSPAWGNRTILAGQSNFGDMVVFDDPITLDNNLHSPPVGRAQGFYFYDRKDVFTAWL
GFSFVFNNSDYRGSINFAGADPLLIKTRDISVIGGTGDFFMARGIATLMTDAFEGEVYFRLRT
DIKLYECY

>XP_002297997.1 pathogenesis-related family protein [Populus trichocarpa]

MEAKRLILALFLLFLLSKSSAFPSRKSRVHKPCKRLVFYFHDIIYNGKNSKNATAAIVGAPAW
GNKTILANQNHFGDLVVFDDPITLDNNLHSAPVGRAQGIYVYDKKEIFTAWLGFSFVFNSTE
HKGSINFAGADPLMNKTRDVSVIGGTGDFIMARGIATLMTDAFEGEVYFRLRVDIQLYECW
